# Genetic structure and seed-mediated dispersal rates of an endangered shrub in a fragmented landscape: a case study for *Juniperus communis *in northwestern Europe

**DOI:** 10.1186/1471-2156-12-73

**Published:** 2011-08-22

**Authors:** An Vanden-Broeck, Robert Gruwez, Karen Cox, Sandy Adriaenssens, Inga M Michalczyk, Kris Verheyen

**Affiliations:** 1 Research Institute for Nature and Forest, Geraardsbergen, Belgium; 2 Laboratory of Forestry, Ghent University, Melle-Gontrode, Belgium; 3 Mobile DNAnalyse, Ebsdorfergrund, Germany

## Abstract

**Background:**

Population extinction risk in a fragmented landscape is related to the differential ability of the species to spread its genes across the landscape. The impact of landscape fragmentation on plant population dynamics will therefore vary across different spatial scales. We quantified successful seed-mediated dispersal of the dioecious shrub *Juniperus communis *in a fragmented landscape across northwestern Europe by using amplified fragment length polymorphism (AFLP) markers. Furthermore we investigated the genetic diversity and structure on two spatial scales: across northwestern Europe and across Flanders (northern Belgium). We also studied whether seed viability and populations size were correlated with genetic diversity.

**Results:**

Unexpectedly, estimated seed-mediated dispersal rates were quite high and ranged between 3% and 14%. No population differentiation and no spatial genetic structure were detected on the local, Flemish scale. A significant low to moderate genetic differentiation between populations was detected at the regional, northwest European scale (PhiPT = 0.10). In general, geographically nearby populations were also genetically related. High levels of within-population genetic diversity were detected but no correlation was found between any genetic diversity parameter and population size or seed viability.

**Conclusions:**

In northwestern Europe, landscape fragmentation has lead to a weak isolation-by-distance pattern but not to genetic impoverishment of common juniper. Substantial rates of successful migration by seed-mediated gene flow indicate a high dispersal ability which could enable *Juniperus communis *to naturally colonize suitable habitats. However, it is not clear whether the observed levels of migration will suffice to counterbalance the effects of genetic drift in small populations on the long run.

## Background

Habitat fragmentation and destruction eventually lead to a reduction in the genetic diversity of plant populations. The consequences of habitat fragmentation are related to the differential ability of plant species to spread their genes across the landscape [1]. Dioecious plant species, with separate male and female plants, appear to have a higher extinction probability compared to cosexual (hermaphroditic and monoecious) plant species (e.g. [2-4]). Separation of the sexes halves the densities of both potential mates and offspring-producing individuals. Furthermore, a dioecious species contributes propagules to fewer sites than a hermaphroditic species with equivalent adult density because the separation of the sexes reduces the density of offspring-producing individuals (i.e. the seed-shadow handicap, reviewed by [3]). This elevated density increases local resource competition thereby reducing each seed's chance of establishing a new plant [3,4]. To overcome these disadvantages, dioecious species require a larger dispersal ability of seeds compared to cosexual species in order to increase their success on the long-term [3].

One example of a locally endangered dioecious species in a fragmented habitat is common juniper (*Juniperus communis *L.). Common juniper is a wind-pollinated, coniferous shrub producing seeds that are primarily dispersed by birds. In northwestern Europe, common juniper occurs on heathlands and calcareous grasslands, which are among the most highly fragmented semi-natural ecosystems in western Europe [5-8]. One of the main threats to common juniper populations is the lack of recruitment from seeds linked to low seed viability. Considerable variation in seed viability was found across European populations [9], which was partly explained by temperature and nitrogen deposition. This in turn could be partly linked to the occurrence of seed predators (insects, mites and/or fungi) and mycorrhizae, respectively. However, the exact processes behind the observed low seed viabilities are not clear yet. One possible explanation for the decline in viable seed production is inbreeding depression caused by increased inbreeding in small and fragmented populations (e.g. [10,11]).

Common juniper has been the subject of a few previous genetic studies (e.g. [5,12-14]). Oostermeijer *et al*. [13] found low population differentiation and high levels of genetic variation in 12 Dutch common juniper populations by studying allozymes. High levels of genetic diversity were also found in 23 common juniper populations from Central-Europe [12]. However, in a study of 19 common juniper populations from Ireland based on nuclear microsatellite data and on chloroplast single nucleotide polymorphisms, Provan *et al*. [14] suggested that, despite dioecism and wind pollination, gene flow is restricted in fragmented landscapes, particularly over larger geographic distances. Also Van Der Merwe *et al*. [5] found that there is little effective gene flow in common juniper based on a study using AFLPs on eight populations from England and Wales. The former studies discussed gene flow based on variants of Wright's F_ST _(such as Φ_PT _/Φ_ST_, G_ST_), a standardized measure of the genetic variance among populations extrapolated from genetic frequency data [15]. This indirect measure of gene flow gives an estimate of historical gene flow (e.g. gene flow that occurred several generations ago predating human-mediated habitat fragmentation), rather than contemporary gene flow (e.g. gene flow that has occurred say, during the last 50 years which can be considered recent for long-lived species) [16]. There is often limited quantitative information to be gained about on-going dispersal from this approach [16].

An alternative for detecting effective gene flow is a population assignment test for individuals based on a large number of polymorphic markers, such as amplified fragment length polymorphisms (AFLPs) (e.g. [17-19]). The idea behind assignment tests is to use individual genotypes to assign individuals to populations or clusters. Given a set of populations, and the allele frequencies of those populations, the likelihood of a given individual's genotype in the population in which it was sampled is calculated and compared with its likelihood in the other populations in the set. An individual is assigned to the population for which it has the highest likelihood. A major advantage of assignment methods is that populations do not have to be sampled exhaustively [20].

**Figure 1:**
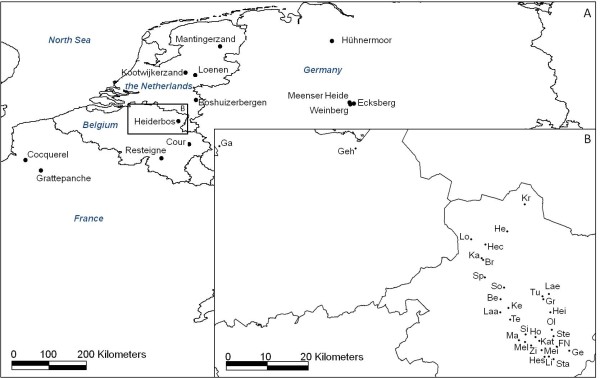
**Locations of the common juniper populations sampled**.

In this study, we used assignment tests to investigate seed-mediated dispersal of common juniper in a fragmented landscape across northwestern Europe. If seed-mediated gene flow is restricted, we expect practically all the sampled individuals to be assigned to the population in which they were sampled. Furthermore, we determined the genetic structure and the genetic diversity of common juniper at two spatial scales: at the regional scale across northwestern Europe and at the local scale across Flanders (northern Belgium) (Figure 1, Table 1). Identifying the spatial scale at which genetic differentiation can be detected will help to determine the factors that cause genetic structure. At the local scale, limited pollen and seed dispersal have been identified as the main forces causing genetic structure [21]. At the larger scales, genetic structure has been attributed to historical factors and isolation-by-distance [22]. We also investigated the correlation between genetic diversity and levels of inbreeding with population size, seed viability and age structure. Finally, we propose conservation strategies for common juniper in northwestern Europe.

**Table 1:** Characteristics of the sampled populations and sample sizes

*Northwestern European J. communis populations*								
**Country**	**Population**	**Code**	**N_c_**	**Area (ha)**	**Density (N /ha)**	**No. of shrubs sampled for seed cones**	**No. of individuals sampled for AFLP**	**No. of unique genotypes**

Belgium	Cour	Cou	55	1	55	6	9	9
Belgium	Heiderbos*	Hei	6874	10	687	27	31	30
Belgium	Resteigne	Res	386	8	48	17	24	24
France	Cocquerel	Coc	1823	4	456	11	19	18
France	Grattepanche	Gra	1108	5	222	18	32	32
Germany	Hühnermoor	Hue	425	6.6	64	22	34	33
Germany	Ecksberg	Eck	50	0.5	100	9	12	12
Germany	Meenser Heide	Mee	60	1.5	40	7	14	14
Germany	Weinberg	Wei	47	1	47	10	13	12
The Netherlands	Boshuizerbergen	Bos	4500	10	450	21	35	35
The Netherlands	Kootwijkerzand	Koo	250	6	42	10	14	12
The Netherlands	Loenen	Loe	200	33.8	6	10	21	21
The Netherlands	Mantingerzand	Man	5000	77	65	18	34	34
*Total*						*186*	*292*	*286*
***Flemish J. communis locations***								

Country	Population /Location	Code	N_c_	Area (ha)	Density (N /ha)		No. of individuals sampled for AFLP	No. of unique genotypes

Belgium	Heiderbos*	Hei	6874	10	687		57	57
Belgium	Kattevennen	Kat	820	12.9	68		26	26
Belgium	Hesselberg	Hes	129	4.8	269		27	26
Belgium	Spiekelspade	Sp	38				4	4
Belgium	Het Laer	La	26				10	10
Belgium	Melberg	Mel	26				8	8
Belgium	Turfven	Tu	24				1	1
Belgium	Kamert	Ka	21				6	6
Belgium	Olenderheibos	Ol	13				6	6
Belgium	Zillebos	Zi	9				5	5
Belgium	Heesakkerheide	He	8				2	2
Belgium	Kruisheirenklooster	Kr	8				2	2
Belgium	Zutendaal	Zu	6				2	2
Belgium	Sintmartensberg	Si	5				2	2
Belgium	Bergbos	Be	4				1	1
Belgium	Brand	Br	4				2	2
Belgium	De Maten	Ma	4				2	2
Belgium	Gebrande heide	Ge	4				1	1
Belgium	Ganzeven	Ga	3				1	1
Belgium	Pijnven Lommel	Lo	3				2	2
Belgium	Kelchterhoeve	Ke	2				2	2
Belgium	Meibos	Mei	2				2	2
Belgium	Pijnven Hechtel	Hec	2				1	1
Belgium	De Teut	Te	1				1	1
Belgium	Grote Heide	Gr	1				1	1
Belgium	Hoogzij	Ho	1				1	1
Belgium	Laambeekvallei	La	1				1	1
Belgium	Lietenberg	Li	1				1	1
Belgium	Sonnis	So	1				1	1
Belgium	Stalkerheide	Sta	1				1	1
Belgium	Steleven	Ste	1				1	1
Belgium	Turnhout	Tur	1				1	1
*Total*							*181*	*180*

N_c_: estimated census population size, Area (ha): area covered by the population, Density: estimated population density. * The Flemish population Heiderbos was analysed twice (in 2005 for the sampling on the northwestern European scale and in 2008 for the sampling on the Flemish scale).

## Results

### AFLP error rate and reproducibility

After scoring 101 AFLP markers for the total dataset of the Flemish samples, 7 markers were discarded which resulted in a final dataset of 94 polymorphic markers. Based on the replicates, 274 differences were observed of 2162 phenotypic comparisons (i.e. 23 samples with duplicates typed for 94 alleles), giving an error rate of 12.6% (i.e. an average intra-individual band difference of 11.8 bands). The error rate at the allele level within a gel run was 3.2%, the other 9.4% of the error rate was due to variability between gels. For the assignment of replicates in the cluster analysis, 44 out of 46 fingerprints from the 23 replicated samples were correctly assigned as 'sisters'. Two fingerprints belonging to the same replicate pair were not positioned adjacent to each other but showed a higher genetic similarity to an other sample (collected in an other population) compared to the genetic similarity with its 'sister'-individual. The number of band difference between these two, not correctly assigned fingerprints was 15 and both replicate pairs were grouped in the same cluster (results not shown). The mean pairwise inter-individual genetic distance was considerably higher than the error rate; 35.1% (standard deviation S.D.: 0.05%) and 35.6% (S.D.: 0.02%) for the samples collected on the Flemish scale and on the northwestern European scale, respectively. The simulation and re-assignment procedures implemented in AFLPOP, resulted in an assignment success of the simulated genotypes above 91% (mean 95%) in all the 11 analysed populations, indicating that the probability of misassignments was low (< 9%).

### Habitat fragmentation and seed-mediated dispersal

**Figure 2:**
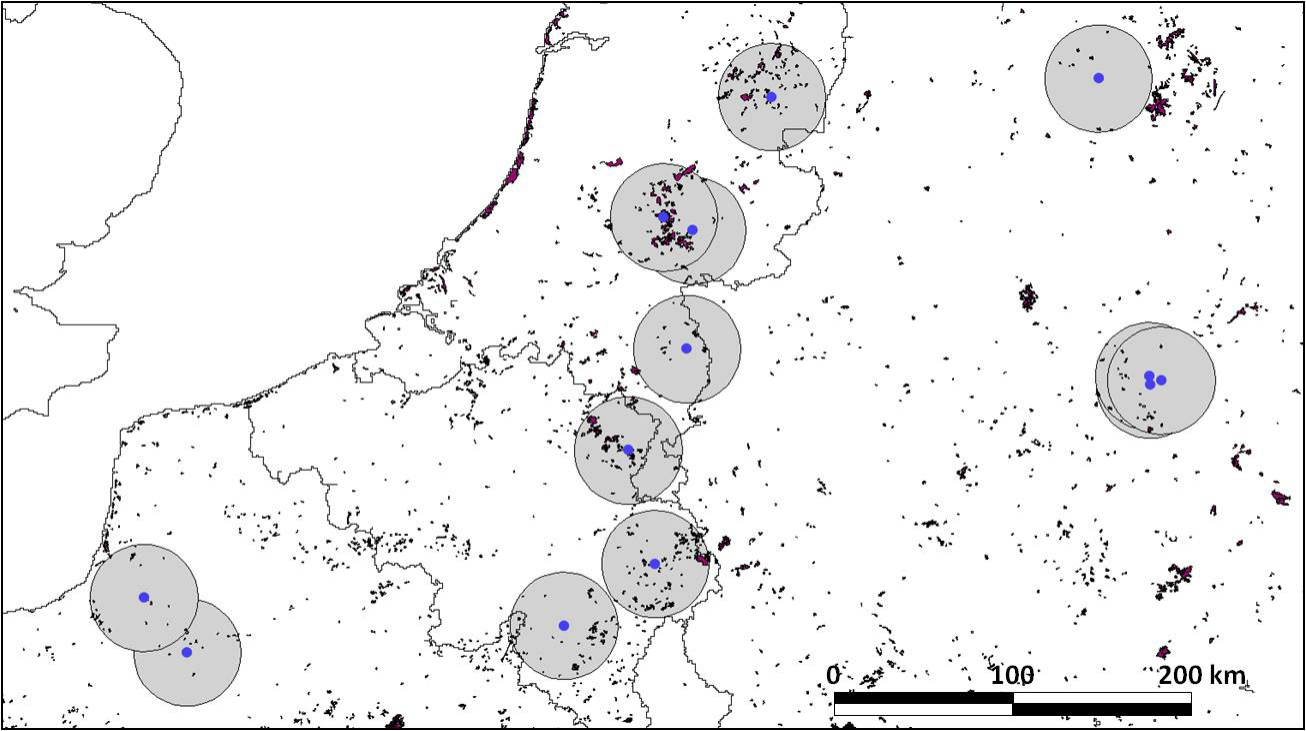
**Distribution of land cover area suitable as habitat for *Juniperus communis***. A 30 km buffer zone is indicated surrounding the sampled populations. (Source: CORINE LAND COVER 2006, version 13 (02/2010), European Environment Agency, http://www.eea.europa.eu/).

In northwestern Europe, considering a land cover area of 1.4E + 7 ha, the estimated proportion of suitable habitat for *Juniperus communis *is 1%. Within a 30 km radius buffer zone surrounding each sampled population, the estimated proportion of suitable habitat for *Juniperus communis *ranged from 0.27% (population Hühnermoor) to 6.75% (population Kootwijkerzand) with a mean of 2.18% (Figure 2).

**Figure 3:**
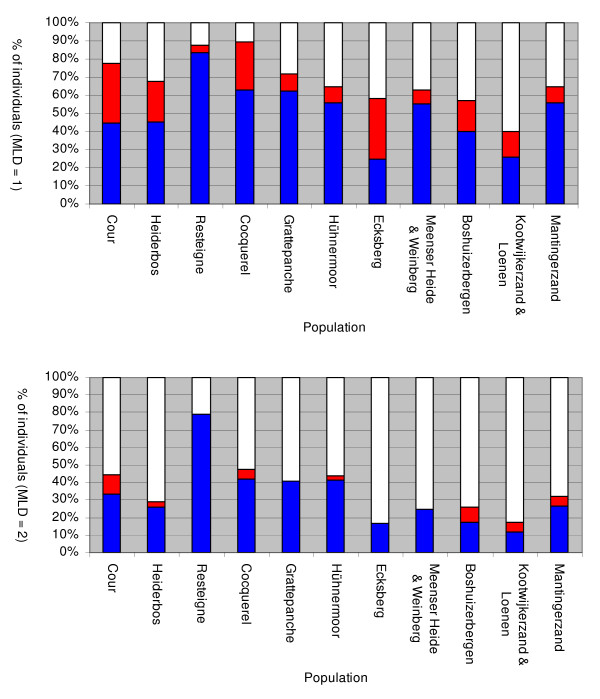
**Individual assignment conducted in northwestern European populations of common juniper**. Results under minimal log-likelihood difference (MLD) set to 1 and 2, respectively. Analysis include individuals from 13 northwestern European common juniper populations and is based on 104 polymorphic AFLP markers. Blue bars represent individuals assigned to their putative population, red bars represent individuals assigned to a population different from the putative population (i.e. migrants); white bars represent individuals not confidently assigned to any of the given populations.

The analysis of the assignment tests indicate that forty-two individuals (14%) were at least 10 times more likely to originate from a population different from the sampling site. Whereas 11 shrubs (3%) seemed at least 100 times more likely to originate from a population different from the sampling population, indicating the presence of migrant genotypes in the set of analysed populations. From the latter 11 plants defined as outlier genotypes (minimum log-likelihood difference (MLD) = 2), six, two and three assumed immigrant genotypes belong to height classes 1 - 2 m, 2 - 3 m and > 3 m, respectively. Assignment tests conducted on the AFLP genotypes of the sampled 292 individuals confidently allocated each of 191 (65%) and 106 (36%) individuals to a population when MLD was set to 1 and 2, respectively (Figure 3). Most individuals that could be confidently allocated were assigned to the spatial population from which they were sampled: 149 (78%) and 95 (89%) for MLD set to 1 and 2, respectively. A high proportion of individuals (45% and 64% for MLD set to 1 and 2, respectively) remained unassigned because there was no single likelihood that met the criteria of the assignment test. However, for half of the unassigned individuals (52 individuals), the population from which they were sampled had the highest log-likelihood of allocation, suggesting that this was the source population.

### Genetic structure

Genetic structure on the northwestern European scale was inferred from 104 polymorphic AFLP markers and 292 shrubs representing 286 unique genotypes (Table 1). There was a moderate but statistically significant genetic differentiation between the 13 populations on the northwestern European scale (Φ_PT _= 0.100, p = 0.001), with a large amount of genetic variation found within populations. Pairwise values for population differentiation (Φ_PT_-values) were also significant (p-values ≤ 0.05) except for the German populations Meenser Heide and Weinberg (Φ_PT _= 0.006, p = 0.3) (Table 2).

**Table 2:** Pairwise population differentiation estimates

	Bos	Coc	Cou	Eck	Gra	Hei	Huh	Koo	Loe	Man	Mee	Res	Wei
Bos	-	0.001	0.001	0.001	0.001	0.001	0.001	0.001	0.017	0.001	0.001	0.001	0.001
Coc	0.129	-	0.001	0.004	0.001	0.001	0.001	0.001	0.001	0.001	0.001	0.001	0.004
Cou	0.106	0.189	-	0.001	0.001	0.001	0.001	0.001	0.002	0.001	0.001	0.001	0.001
Eck	0.111	0.040	0.124	-	0.001	0.001	0.001	0.001	0.001	0.001	0.006	0.001	0.001
Gra	0.078	0.091	0.086	0.076	-	0.001	0.001	0.001	0.002	0.001	0.001	0.001	0.002
Hei	0.042	0.127	0.110	0.107	0.072	-	0.001	0.001	0.002	0.001	0.001	0.001	0.001
Huh	0.048	0.155	0.120	0.128	0.114	0.057	-	0.001	0.001	0.001	0.001	0.001	0.001
Koo	0.105	0.177	0.131	0.150	0.085	0.114	0.143	-	0.005	0.001	0.001	0.001	0.001
Loe	0.024	0.103	0.074	0.075	0.049	0.036	0.066	0.050	-	0.005	0.001	0.001	0.001
Man	0.069	0.144	0.125	0.130	0.075	0.070	0.097	0.065	0.022	-	0.001	0.001	0.001
Mee	0.090	0.049	0.141	0.038	0.075	0.107	0.129	0.138	0.073	0.121	-	0.001	0.294
Res	0.127	0.203	0.068	0.153	0.132	0.129	0.156	0.176	0.105	0.174	0.166	-	0.001
Wei	0.120	0.059	0.182	0.067	0.080	0.132	0.165	0.173	0.087	0.123	0.006	0.181	-

Below diagonal: population pairwise estimates of Φ_PT_- values of 13 common juniper populations sampled in northwestern Europe. Above diagonal: probability values based on 999 permutations. Negative pairwise Φ_PT _- values are converted to zero. Population codes: Bos: Boshuizerbergen, Coc: Cocquerel, Cou: Cour, Eck: Ecksberg, Gra: Grattepanche, Hei: Heiderbos, Huh: Hühnermoor, Koo: Kootwijkerzand, Loe: Loenen, Man: Mantingerzand, Mee: Meenser Heide, Res: Resteigne, Wei: Weinberg.

**Figure 4:**
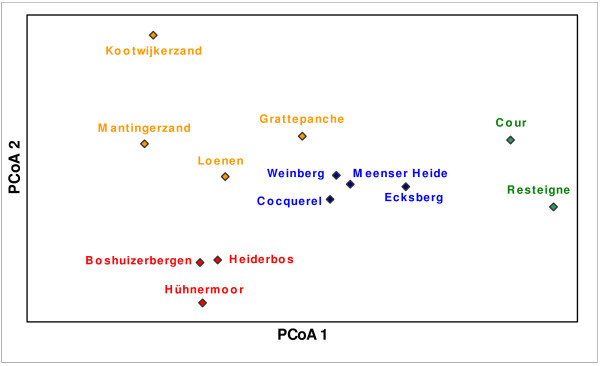
**Plot of the two first axes of the Principal Co-ordinate Analysis (PCoA) colored by Bayesian cluster allocation**. Analysis include 13 northwestern European common juniper populations and is based on 104 polymorphic AFLP markers (PCoA1 = 35.71%, PCoA2 = 22.10%). Each population is assigned by one of the four clusters found by a Bayesian mixture clustering approach.

Clustering on the population level based on a Principal Co-ordinate Analysis (PCoA) and on a Bayesian modelling approach is presented in Figure 4. The Bayesian clustering approach revealed highest posterior probabilities when the number of clusters was equal to four. In general, both the Bayesian approach and the PCoA grouped geographically nearby populations within the same genetic cluster. This is confirmed by a weak but significant isolation-by-distance effect (r _xy _= 0.123, p = 0.01). Although, for some populations, the genetic data did not cluster consistently according to their geographical locations. This was the case for the French populations Grattepanche and Cocquerel and the German population Hühnermoor.

Genetic structure on the local, Flemish scale was inferred from 94 polymorphic AFLP markers and 181 shrubs representing 180 unique genotypes (Table 1). On the local scale, we observed no genetic differentiation between the three populations containing more than 100 individuals; Heiderbos, Kattevennen and Hesselberg (Φ_PT _= 0.00, p > 0.5). Pairwise values for population differentiation were not significant (all Φ_PT _-values p > 0.2). Also, when considering all 180 Flemish genotypes analysed (i.e. including also locations with less than 100 shrubs), no structure according to the population of origin or geographic location was apparent on a PCoA (results not shown). With the Bayesian approach, highest posterior probabilities were obtained with all 180 genotypes located in the same cluster. On the local, Flemish scale, we found no evidence for the isolation-by-distance hypothesis with the Mantel test (r _xy _= 0.052, p = 0.076). Consistently, no spatial genetic structure was found with SPAGeDi 1.3 by regressing pairwise kinship coefficients against pairwise geographical distances (slope of the regression with ln (distance) b = 0.0012, p = 0.61).

### Genetic diversity

High variation was recorded at AFLP loci with a mean gene diversity (*Hj*) of 0.391 and 0.368 for the European and the Flemish populations, respectively. Genetic diversity statistics based on the AFLP markers for the northwestern European as well as for the three Flemish populations are given in Table 3. The same statistics are grouped per height class in Table 4. Individuals with a height less than 1 m showed a lower percentage of AFLP band polymorphism compared to higher shrubs (Table 4). However, there was no evidence for a decrease in genetic diversity in terms of band richness (*Br*) or gene diversity (*Hj*) in these younger individuals compared to the older ones within each population (results not shown), nor for the AFLP data pooled over all populations (Table 4). For the northwestern European populations, the average inbreeding coefficient was higher for the younger individuals than for the older ones (Table 4), although the difference was not significant (p = 0.106). We found no significant effects of population size on genetic diversity measures (all p-values > 0.05). Average percentages of filled seeds and of seed viability per shrub for the populations sampled on the northwestern European scale were low and ranged from 1.73 to 18.98 and from 0.10 to 5.49, respectively (Table 3). We found no significant correlation between seed viability, filled seeds and any genetic diversity measures (all p-values > 0.1). Also no correlation was found between seed viability and population size (p = 0.104).

**Table 3:** Seed characteristics and genetic diversity statistics calculated from AFLP data for 13 northwestern European and three Flemish common juniper populations

	# G	# loc	PPL	Br	Hj (S.E.)	F_IS_	Filled seeds (%)	Seed viability (%)
*Northwestern European J. communis populations*								
Cour	9	104	73	1.73	0.409 (0.012)	0.27	4.41	0.10
Heiderbos*	30	104	100	1.85	0.337 (0.014)	0.42	15.28	5.49
Resteigne	24	104	90	1.65	0.343 (0.013)	0.46	9.47	5.49
Cocquerel	18	104	98	1.93	0.364 (0.012)	-0.06	2.70	0.44
Grattepanche	32	104	100	1.94	0.386 (0.011)	0.04	1.73	0.58
Hühnermoor	33	104	94	1.75	0.331 (0.014)	0.47	11.95	4.58
Ecksberg	12	104	96	1.94	0.389 (0.012)	0.00	11.45	3.89
Meenser Heide	14	104	87	1.82	0.363 (0.013)	-0.02	18.98	2.76
Weinberg	12	104	90	1.86	0.371 (0.013)	0.10	10.02	4.92
Boshuizerbergen	35	104	99	1.75	0.344 (0.012)	0.46	3.80	1.50
Kootwijkerzand	12	104	98	1.93	0.347 (0.013)	-0.14	5.21	0.48
Loenen	21	104	98	1.90	0.363 (0.011)	0.22	6.69	2.32
Mantingerzand	34	104	87	1.91	0.346 (0.013)	0.02	2.70	0.14
*Flemish J. communis populations*								
Heiderbos*	57	94	100	1.86	0.359 (0.012)	0.37	NA	NA
Kattevennen	26	94	98	1.86	0.382 (0.011)	0.41	NA	NA
Hesselberg	26	94	96	1.83	0.364 (0.013)	0.33	NA	NA

# G: number of genets typed with AFLP markers. # loc: number of AFLP loci. PPL: percentage polymorphic loci at the 5% level corrected for sample size. Br: band richness corrected for sample size. Hj (S.E.): expected heterozygosity or Nei's gene diversity and its standard error. F**_IS_**: average inbreeding coefficient. Filled seeds (%): the average percentage of filled seeds per shrub. Seed viability (%): the average percentage of viable seeds per shrub. *The Flemish population Heiderbos was analysed twice (in 2005 for the sampling on the northwestern European scale and in 2008 for the sampling on the Flemish scale).

NA: not assessed.

**Table 4:** Genetic diversity statistics per height class calculated from AFLP data for 13 northwestern European and three Flemish common juniper populations

Height classes	# G	# loc	PPL	Br	Hj (S.E.)	F_IS_
Northwestern European *J. communis *						
< 1 m	48	104	98	1.98	0.376 (0.011)	0.32
1 m - 2 m	73	104	99	1.98	0.368 (0.011)	0.14
2 m - 3 m	72	104	99	1.99	0.360 (0.011)	0.19
> 3 m	90	104	99	1.97	0.364 (0.011)	0.24
Flemish *J. communis *(populations Heiderbos, Kattevennen, Hesselberg)						
< 1 m	8	94	84	1.84	0.391 (0.012)	0.34
1 m - 2 m	32	94	97	1.84	0.381 (0.011)	0.22
2 m - 3 m	31	94	97	1.81	0.334 (0.014)	0.55
> 3 m	38	94	100	1.81	0.377 (0.011)	0.08

# G: number of genets analysed with AFLP markers. # loc: number of AFLP loci. PPL: percentage polymorphic loci at the 5% level corrected for sample size. Br: band richness corrected for sample size. Hj (SE): expected heterozygosity or Nei's gene diversity and its standard error. F_IS_: average inbreeding coefficient.

## Discussion

### AFLP error rates and reproducibility

The observed error rates are higher than those generally reported for AFLP data sets (2% to 5%; e.g. see [23]) but lower than the one reported by Mende *et al*. [24] (19%) and within the range of the ones reported by Holland *et al*. [25] (between 6% and 18%) and Storme *et al*. [26] (8%). However, care must be taken when comparing the error rate between different AFLP studies. Error rates are affected by the way they are calculated, the level of divergence among the studied individuals, the number of individuals in the dataset, the technical aspects of generating the profiles (e.g. PCR errors, slab gel versus capillary electrophoresis) and the scoring process (i.e. manual scoring, semi-automated or automated scoring procedures) ([25,27]). Furthermore, there is a trade-off between the number of loci used to record the AFLP information and the accuracy of the dataset (i.e. the error rate) ([25,28,29]). Tolerance of a higher error rate result in the retention of more loci which generally leads to higher resolution of the dataset ([28,29]). Bonin *et al*. [30] concluded that the maximum tolerable error rate depends on the specific aim and circumstances of the study. Based on the relative high level of divergence between the individuals in the total dataset (mean 35%), the assignment accuracy of the replicate pairs in the cluster-analysis (95%) and the relative low probability of misassignments in the simulated re-assigment procedure (< 9%), we conclude that our dataset contains a significant genetic signal in excess of the error rate for the study of the genetic diversity, population genetic structure and seed-mediated dispersal rates.

### Habitat fragmentation and seed-mediated dispersal

The analysis of the CORINE 2006 land cover data indicate a highly fragmented habitat for *J. communis *in northwestern Europe. Habitat fragmentation and habitat loss usually decrease the probability that migrant seeds will find suitable sites for establishment. By providing insight into seed-mediated dispersal rates as a direct estimate of patterns of gene flow, our results expand on previous work (e.g. [5,12-14]). We are aware that we did not sample all the potential source populations. Our aim was not to allocate individuals to the sampled populations, but to estimate migration rates by identifying immigrants; individuals that originate from somewhere else than where they were sampled (e.g. see [17]). The results of this study indicate that gene flow might not be so restricted as previously thought (e.g. [5,14]). The assignment test, based on the AFLP-genotypes of individuals revealed that 42 individuals (14%) of all 292 individuals sampled in the northwestern European populations showed a genetic resemblance of 10 times higher to another population than the one from which it was sampled. Furthermore, 11 individuals (3%) of all individuals sampled displayed a resemblance to other populations that was at least 100 times higher. Consequently, we conclude that at least between 3 and 14% of all individuals from the sampled northwestern European populations, originated from seeds from outside the population from which they were sampled. We interpret these confident allocations of individuals to other source populations as a consequence of seed dispersal events, as it is unlikely that effective pollen flow could generate such a high genetic resemblance with another population (cf. [17,19]). It has to be mentioned that we can not exactly determine the year of the assumed seed-mediated dispersal events as we did not define the exact age of each individual shrub. However, given that the maximum shrub life-span is estimated to about 100 years and that the majority of the genetic outliers has a shrub height smaller than 2 meter, it is unlikely that the recruitment of all these genetic outliers predate the fragmentation process which started about 100 years ago. It should also be noted that we could not quantify the exact distance of the seed dispersal events. Although information can be found for each northwest European country on the distribution of common juniper, data about the estimated census population size, density or fragment size of the populations is generally lacking. Therefore we were not able to quantify the degree of isolation of the studied populations and the distances covered by the seed-migration events. The seed-mediated dispersal rates reported in this study are in the range of the long-distance seed-dispersal events reported from assignment tests in fragmented populations of *Banksia hookeriana *(6.8%) [19], of which the seeds are primarily dispersed by gravity, but much lower than that reported for the frugivore-dispersed trees *Myrtus communis *(20% - 22%) [17] and *Fagus sylvatica *(27%) [31]. In a study of common juniper populations on a Mediterranean mountain in southeast Spain, Garcia [32] found that thrushes spent a substantial proportion of their time in junipers feeding on cones. This results in large accumulations of seeds below mother plants after disperser activity [32]. It is speculated before that dioecious species experience reduced extinction rates when associated with woody growth form and biotic dispersal via fleshy fruits [2,3]. Although the majority of the seeds may be dispersed within the population, it is plausible that a significant proportion of dispersal events between populations of common juniper is caused by birds tracking fruit resources across the landscape. Although we are not able to assess the distance covered by the seed dispersal events with the available data, our findings provide evidence of seed-mediated among-populations gene flow in common juniper within the study area. However, moderate levels of gene flow by seed dispersal may not be sufficiently high to counterbalance the effects of genetic drift and inbreeding in remnant common juniper populations located in a fragmented landscape. Further research should explore effective mating patterns and the precise spatial scale and rates at which gene flow occurs.

### Population genetic diversity and structure

Population genetics theory predicts that habitat fragmentation increases genetic differentiation among populations because of increasing spatial isolation between patches and individuals (e.g. [1]). At the local, Flemish scale, the three populations studied showed no genetic differentiation and no spatial genetic structure. This may be attributed to the fact that at this local scale, seed and pollen dispersal may have homogenized allele frequencies. At the larger geographical scale across northwestern Europe we found a significant low to moderate degree of population differentiation (Φ_PT _= 0.103). Our results are in agreement with those reported for outcrossing, wind-pollinated gymnosperm species [33,34], usually characterized by high genetic diversity within populations and low to moderate population differentiation. Based on allozyme loci and on a more local scale, Oostermeijer and Knegt [13] found a much lower level of population differentiation among 12 common juniper populations in the Netherlands (F_ST _= 0.026). This is probably linked to the fact that allozymes show lower levels of polymorphism compared to AFLP markers and may, in contrast to neutral AFLP markers, experience the selective force of the environment. Similar to this study, Provan *et al*. [14] also found a low to moderate degree of population differentiation between 19 populations of common juniper in Ireland based on nuclear microsatellite markers (Φ_PT _= 0.0957) but a much higher degree of differentiation between the same populations based on chloroplast markers (Φ_PT _= 0.249). The latter is explained by the action of genetic drift on the smaller effective population size of the uniparentally (particular parternally) transmitted, haploid chloroplast genome [14].

In general, we found a clear but weak association between the genetic clustering and the geographical location of the common juniper populations studied; neighbouring populations were generally also genetically related. Exceptions to this general pattern were the French populations Cocquerel and Grattepanche and the German population Hühnermoor, which did not cluster consistently to their geographical location. The weak geographic structuring of genetic diversity on the northwestern European scale was confirmed by a weak isolation-by-distance signal. In contrast, Michalczyk *et al*. [12] found no isolation-by-distance effects and no meaningful geographic genetic structure in 23 common juniper populations sampled throughout Central-Europe. However, in Great Britain, Van Der Merwe *et al*. [5] found a genetic clustering of geographically proximal populations. A coherent genetic clustering of geographically nearby populations was also the case for the common juniper populations studied in Ireland although, similar to our study, there were some exceptions [14].

The geographic patters of genetic diversity observed in this study may be explained by the patterns of genetic diversity in the original metapopulation prior to fragmentation. Like Michalczyk *et al*. [12] hypothesized for Central-Europe, the northwestern populations could also originate form a large, continuous population characterized by high levels of genetic diversity, that was relatively recently subject to fragmentation. Historical high rates of pollen and seed-mediated gene flow could have maintained the genetic cohesion of the populations. The fairly recent loss of individuals and populations may have been more or less random with respect to gene content. The low to moderate levels of population differentiation may indicate that genetic drift has provoked weak but significant fluctuations in allele frequencies between populations after habitat fragmentation. The results of this study may provide further support for the northern refugia hypothesis, a recent controversial proposal, suggesting that trees were distributed much more widely in Europe during the last glacial maximum (LGM, 21 ka BP) than previously thought [35]. This hypothesis is in contrast to the general understanding of the last few decades that during several glacial maxima, most temperate tree species were restricted to refuge areas in the Balkan, Italian and Iberian peninsulas (the southern refugia hypothesis) [36,37]. Based on the lack of a strong isolation-by-distance signal and the absence of genetic lineages or coherent geographical patterns of genetic diversity that could be traced back to southern refugia, we assume that the cold-adapted, drought-tolerant common juniper could have survived throughout northwestern Europe in scattered and diffuse habitats during the LGM (21 ka BP). This is also hypothesized for common juniper in Central-Europe by Michalczyk *et al*. [12]. Fossil records support the hypothesis that juniper could have survived the LGM in Europe [12,38] in contrast to tree species like oak and poplar that prefer warmer conditions and were squeezed into lower latitudes [39,40]. There is also evidence from a study on molecular dating, based on cpDNA phylogeny conducted over the Northern Hemisphere, that the genus *Juniperus *has an extremely long history in Europe [41]. *Juniperus *may already have been distributed across Europe during the earliest Tertiary period (65 million years ago), at the start of its history, and appears to have colonized Asia, Africa and America from Europe via intercontinental land bridges [41].

We observed high levels of within-population genetic diversity (in terms of band richness and percentage polymorphic loci) in all common juniper populations studied. This result is in line with previous genetic studies of common juniper employing different marker systems (nuclear microsatellites, AFLPs, isozymes) [5,12-14]. While changes in genetic diversity following a decrease in population size can take a number of generations to become apparent, which for trees may take many decades, this may not be the case for inbreeding (i.e. mating between relatives) (e.g. [42,43]). Although not statistically significant (p = 0.106), a trend towards a higher inbreeding coefficient was found in the younger individuals (height < 1 m) compared to the older ones (height > 1 m) on the northwestern European scale. It has to be mentioned that, due to a extremely low recruitment in the studied populations, the number of seedlings sampled was low and consequently limited the power of this study.

Although it is suggested in previous studies that the extremely low seed viability is likely not linked to neutral genetic diversity [9], this is the first study that investigates this relationship. We detected no correlation between inbreeding coefficient or any other genetic diversity parameter, and seed viability across the studied populations in northwestern Europe. Also, in accordance with the results of Oostermeijer and De Knegt [13] we found no correlation between population size and any genetic diversity parameter. Reduction of the formerly widespread dry heathland habitats of common juniper populations in northwestern Europe mainly occurred since the beginning of the 20^th ^century [13,44]. At present, most populations in northwestern Europe are dominated by mature and old individuals of 40 - 100 years and suffer from a lack of natural regeneration [13,14,44]. Hence, they were established before or shortly after habitat fragmentation occurred. This implies that to date, the relict populations of northwestern Europe retain the high proportion of genetic diversity that was likely present when the populations were large and panmictic. The ability of common juniper to reproduce clonally, via resprouting and vegetative spread, may also buffer the genetic effects of fragmentation as a result of extending the time between generations (e.g. [45]). However, we found the vegetative reproduction of common juniper in the populations studied to be rather limited.

## Conclusions

Our study contributes new data to the growing evidence that seed-mediated dispersal of dioecious shrubs and trees in fragmented landscapes is substantially higher than previously thought. The potential for seed-mediated dispersal among populations in a fragmented landscape could enable common juniper to naturally colonize suitable habitats. Nevertheless, management strategies that exclusively focus on seed dispersal would not guarantee recruitment in common juniper because of the extremely low levels of seed viability. These seems to be currently the main threat to common juniper populations in northwestern Europe (e.g. [9]). The low levels of seed viability found within populations are not correlated to the levels of neutral genetic diversity. The exact mechanisms behind the lack of recruitment should be clarified by further research. In the meanwhile, we suggest two main conservation actions; firstly, static *ex situ *conservation of the present high levels of genetic diversity by the establishment of gene banks, and secondly, rejuvenation of existing, small populations skewed to old plants by restoration planting with young individuals grown from cuttings which should enhance the seed production of the populations [46]. Given the high levels of local genetic diversity it is advisable to use locally sourced plant material for restoration and rejuvenating on projects in order to avoid potential outbreeding depression.

## Methods

### Study species

*Juniperus communis *L. (Cupressaceae) is a diploid, dioecious, wind-pollinated, woody shrub or small tree. The distribution range of common juniper basically covers the entire Northern Hemisphere [47]. It is the most widespread conifer taxon worldwide and known to have a broad ecological amplitude. Female individuals bear cones that ripen fully in the autumn of the second or third year of development and contain 1 - 3, rarely 4 seeds. Seeds are mainly dispersed by birds, especially thrushes (*Turdus *spp.) and common juniper does not produce a long-term persistent seed bank [48]. Lifespan is estimated to be about 100 years, although exceptionally individuals reach over 200 years [46]. Genets with clonal shoots, however, may readily exceed this age. Common juniper habitats have been accorded a legal protection status in Europe (EU Habitat Directive, Annex I, code 5130) and despite its wide distribution, the species is on the Red List in several European countries (e.g. the Netherlands [49], the UK [50] and Belgium [51,52]). For more information about the species, we refer to Thomas *et al*. [53].

### Sample collection

In 2005, we sampled 13 natural common juniper populations along a north-south transect from north-Germany and northern Netherlands to north France, and along an east-west transect from northwest France to northwest Germany (Figure 1). Populations located along the transect were selected for sampling when they contained at least 100 individuals and were presumed natural. A population was presumed natural if there was evidence based on historical topographic maps or if personal communications with local people and nature conservationists revealed that the population was at the site for many centuries. When no populations with at least 100 individuals were present, presumed natural populations with less individuals were included. The sampled populations included three populations in northwest France, three populations in Belgium, four in the Netherlands and three populations in northwest Germany. The populations occurred on heathlands or calcareous grasslands, both strongly fragmented habitats in the study area [7,8]. For each population, the census population size was estimated based on the point-centred quarter method (PCQ), which is a plotless sampling method to estimate the population density [54]. Therefore, in each population, one to three random sampling points were laid out depending on the size of the population. In each of the four quarters around the sampling points, distances were measured to a maximum of four trees closest to this point; one for each of four height classes (when available): < 1 m, 1 - 2 m, 2 - 3 m, > 3 m. These height classes broadly reflect the following development classes of the tree: seedlings, young plants, mature plants and old plants [46]. Fresh needles were collected from the measured trees in each quarter. The needles were dried with silica gel in zip-locked bags until analysis. Next to this, a random sample of ripe cones was collected from the female shrubs at each sampling point, resulting in 6 to 27 plants per population. Table 1 provides information about the populations sampled.

In 2008, we sampled the three natural (defined as above) populations in Flanders that contain more than 100 individuals: the populations Heiderbos, Kattevennen and Hesselberg. The populations occurred on heathlands, a habitat that has become highly fragmented in Flanders [55]. Moreover, we sampled 29 other locations in Flanders with one to 38 relict individuals. The three natural Flemish populations were analysed for genetic diversity statistics while the spatial genetic structure and clonal structure was inferred from Flemish samples collected on all the 32 locations. All sampling locations were located in the east of Flanders (northeast Belgium). They were selected from an earlier full inventory of *Juniperus communis *in Flanders [56]. Again, individuals from different age classes were sampled. The height of the shrubs was recorded as mentioned above. Shrubs were sampled at random at each location since census population sizes often were too small to use the PCQ-method. Information about the sampling sites and their location is given in Table 1 and Figure 1, respectively.

### Seed viability

Per shrub, 10 ripe cones were opened, the number of seeds was counted and filled seeds were exposed to 1% 2,3,5 triphenyltetrazolium chloride (TTC) solution in order to determine the viability of the embryos. Initially colourless, TTC is converted to formazan-red in the presence of living tissue (see e.g. Miller [57] for more details on the method).

### DNA extraction and molecular genotyping

Genomic DNA was extracted from 20 mg of dried needles using the Dneasy Plant Miniprep Kit (Qiagen, Helden, Germany), according to manufacturer's instructions and followed by an additional treatment with 0.4 μg RNAse (Fermentas) at room temperature for 2 min. DNA concentrations were estimated and standardised against known concentrations of λDNA (Fermentas) on 1.5% agarose gels.

AFLP analysis was performed on the northwestern European and the Flemish samples according to Vos *et al*. [58] and Van Der Merwe *et al*. [5] with following modifications. Restriction and ligation were performed in a single step, e.g. 200 ng of genomic DNA was restriction digested using the enzyme combination *PstI *(Fermentas) /*MseI *(Fermentas) and ligated to the *PstI *and *MseI *adaptors. Primer combinations used for the generation of fingerprints were *PstI*-ACT + *MseI*-ACA, *PstI-*ACT + *MseI*-ACC, *PstI*-AGT + *MseI*-ACC and *PstI*-AGT + *MseI*-ACA. Fragment separation and detection took place on a NEN IR^2 ^DNA analyzer (Li-Cor Biosciences) using 36 cm denaturing gels with 6.5% polyacrylamide. IRDye size standards (50 to 700 bp) were included for sizing of the fragments. Fragments within the size range of 75 bp to 677 bp were scored with Saga Generation 2 (Li-Cor Biosciences) as present or absent. Prior to data analysis, monomorphic loci were discarded. Due to the long time period between the analysis of the northwestern European samples and of the Flemish samples, different PCR-machines and fabrication batches of products were used. Hence, following Coart *et al*. [59], the AFLP-data of the northwestern European samples and of the Flemish samples were processed separately. The number of individuals typed with AFLP markers is given in Table 1.

### AFLP error rate and reproducibility

Reproducibility was evaluated on the dataset obtained from the individuals sampled on the Flemish scale using intra- and intergel replicates. 23 samples (12%, according to the recommendations of Bonin *et al*. [23]) were chosen randomly and analysed twice independently starting from the same DNA-extraction. Samples with a profile that was doubtful, for example profiles showing low band intensities, were discarded. We estimated the error rate at the allele level as described by Bonin *et al*. [23] based on the binary matrix obtained for the replicate samples. This error rate is effectively the average Euclidean distance (= 1 - Simple Matching similarity index [60]) between replicate pairs. The error rate was first used to eliminate unreliable markers (markers difficult to score or unstable markers) and to clean up the binary data matrix [30]. Secondly, we recalculated the error rate based on the replicated samples for the final markerset. In order to evaluate this error rate in accordance to the goal of the study, we performed a UPGMA-cluster-analysis based on the Simple Matching similarity index calculated from the binary matrix of the replicated samples using the programme TREECON [61]. We calculated the number of replicate pairs that were correctly assigned (i.e., as 'sister' to one another) in the cluster analysis (e.g. see [25]). We also calculated the mean pairwise inter-individual genetic distance based on the Simple Matching index for all the genotypes from the Flemish dataset and from the northwestern European dataset, and compared this with the mean intra-individual genetic distance (= equivalent to the error term). Furthermore, we implemented the simulation procedure in the programme AFLPOP [62] to investigate the power of the data for the assignment test.

### Habitat fragmentation and seed-mediated dispersal

To estimate the degree of habitat fragmentation in the study area, we mapped the natural habitats suitable for *Juniperus communis *with the programme ArcView version 3.1. (ESRI) and based on CORINE Land Cover 2006 vector data (CLC06) (version 13 - 02/2010, http://www.eea.europa.eu/). CLC06 classifies the European land cover into 44 categories derived from Landsat and SPOT satellite images at a 1:100,000 scale and with a minimum mapping unit of 25 ha [63]. The following land cover types from CLC06 were considered as suitable habitats for *Juniperus communis*: natural grasslands (class 3.2.1), moors and heathlands (class 3.2.2), sclerophyllous vegetation (class 3.2.3) and transitional woodland-shrub (class 3.2.4). The percentage of suitable habitat was calculated for northwestern Europe within an area of 1.4E+7 ha, and within a 30 km radius buffer zone surrounding each sampled population.

Seed-mediated dispersal events were estimated by individual-based population assignment tests using the computer program AFLPOP 1.1 [62]. Because we are aware that we did not sample all the potential source populations, our aim was not to allocate individuals to some of the sampled populations, but to estimate migration rates by identifying immigrants for the populations sampled on the northwestern European scale. First, the likelihood was computed that an individual genotype (G) may be found in each of the candidate populations based on their respective dominant AFLP band frequencies. G is then assigned to the population showing the highest likelihood for G [64]. Given that pollen flow might result in ambiguous assignments when low levels of stringency are used [19], allocation tests were conducted setting the minimum log-likelihood difference (MLD) to 1 and 2. At these MLD = 1, MLD = 2 stringency levels, an assignment to a population is made when the probability of the given assignment is ten or 100 times more likely than the next most probable assignment, respectively. Other settings in the program were: replace zero frequencies by (1/(sample size+1)) and calculate a p-value for each individual's log-likelihood by creating empirical distributions from 1000 randomly generated genotypes based on the presence frequencies of each population. When the p-values for an individual were below a certain warning threshold (< 0.001 in our case) for all candidate populations, it was concluded that the individual did not originate from any of the sampled populations.

Prior to the allocation test, we assessed the power of our dataset for accurate assignment of the real genotypes with the population assignment simulator of AFLPOP 1.1 [62]. The simulator generated 1000 random genotypes based on the observed allele frequencies in each sampled population. Those 1000 simulated genotypes were then blindly reassigned to their most probable population. The simulation process was repeated 10 times to check for the consistency of the results. Because of geographical affinity and small population size we pooled the samples from populations Kootwijkerzand and Loenen, and also the samples from the populations Meenser Heide and Weinberg. The latter populations were also pooled because of statistically non-significant Φ_PT_-pairwise values. This reduces the risk of misassignment due to similar allele frequencies between population pairs.

### Genetic structure

Population genetic structure was analysed based on AFLP data on both spatial scales. Total genetic diversity was partitioned among and within populations by carrying out a hierarchical analysis of molecular variance (AMOVA) on Euclidian pairwise genetic distances [65]. The Φ_PT _analog for F_ST _[66] was calculated based on Euclidian genetic distances, and its significance was determined using the Monte Carlo procedure (999 permutations). Based on these Euclidian pairwise genetic distances a principal coordinates analysis (PCoA) was performed. These analyses were carried out using GENALEX 6.2 [67]. To further identify possible spatial patterns of genetic diversity, the software BAPS 5.3 [68] was used to identify clusters of genetically similar populations using a Bayesian approach. A population mixture analysis was performed for the maximum number of clusters (K) ranging from K = 1 up to K = 15. We ran the cluster analysis ten times in order to test the reproducibility of the results. In order to identify a significant isolation-by-distance effect [69], a Mantel test was performed on pairwise genetic distances and geographic distances. At the local (Flemish) scale, we investigated the existence of a fine scale spatial genetic structure. We plotted and regressed average pairwise kinship coefficient of relatedness for dominant markers [70] against geographical distances with the software SPAGeDi 1.3 [71].

### Genetic diversity

As common juniper can reproduce clonally, we first checked whether the dataset contained similar ramets of the same clone. This was done with AFLPdat [72] by setting the maximum number of differences among identical individuals to 15 bands. The latter was estimated from 23 replicated samples. Further population genetic analyses were restricted to the individuals derived from sexual reproduction (i.e. genets). Genetic diversity statistics were calculated based on AFLP data. We calculated AFLP fragment frequencies with AFLPsurv 1.0 [73] to estimate allele frequencies for each population. This was based on a Bayesian approach with a non-uniform prior distribution of allele frequencies following Zhivotovsky [74], assuming either no, or some deviation (F_IS _= 0.1) from Hardy-Weinberg genotypic proportions according to the outcrossing nature of the species. However, the results based on the different F_IS_*-*values were very similar and therefore, only those based on F_IS _= 0 are presented. Allele frequencies were then used to calculate Nei's gene diversity (*Hj*) and the percentage of polymorphic loci at the 5% level corrected for the sample bias (*PPL*). Furthermore, band richness corrected for the sample bias (*Br*) was computed on the AFLP data with AFLPDIV (first described in [59]). This measure of genetic diversity represents the number of phenotypes expected at each locus (i.e. each scored AFLP fragment) and can be interpreted as an analogue of allelic richness [59].

The level of inbreeding (F_IS_) was estimated from AFLP fragment frequencies using FAFLPcalc [75]. FAFLPcalc uses AFLP frequencies to estimate band frequencies that are used to simulate data with a range of inbreeding coefficients. This approach assumes that half of the individuals in a population are outbred, and that inbred individuals will be more homozygous (exhibit more null phenotypes). Scoring errors and high levels of non-independence between bands can lead to poor results, which is why we compare calculated F_IS _values only among our sample populations and within a sampling year and not to those from other studies.

In order to identify whether habitat fragmentation resulted in a decrease of genetic diversity in younger (height < 1 m) compared to older individuals (height > 1 m) of the common juniper populations, values of Φ_PT _were calculated between the four different development classes by AMOVA based on AFLP data for each population separately. Also, values of Φ_PT _and genetic diversity statistics (*PPL, Br, Hj*) were calculated for pooled population samples per height class within each spatial scale. Average inbreeding coefficients (F_IS_) were compared between young plants (height < 1 m) and older individuals (> 1 m height) of the common juniper populations sampled on the northwestern European scale by a t-test. Finally, we used Spearman rank correlations to identify possible relationships between AFLP-based genetic diversity measures (*PPL, Br, Hj *and F_IS_) and population characteristics (population size, population density, % filled seeds and % viable seeds).

## Authors' contributions

AV, RG, SA, KV designed the research. RG, SA collected field data. AV, KC participated in the molecular genotyping. AV, KC, KV analyzed the data. AV drafted the manuscript. IM helped to draft the manuscript. All authors discussed the results and commented on the manuscript. All authors read and approved the manuscript.

## References

[B1] Young A Boyle T Brown T The population genetic consequences of habitat fragmentation for plants Trends in Ecology & Evolution 1996 11 413 418 10.1016/0169-5347(96)10045-8 21237900

[B2] Vamosi JC Vamosi SM The role of diversification in causing the correlates of dioecy Evolution 2004 58 723 731 15154548 10.1111/j.0014-3820.2004.tb00405.x

[B3] Heilbuth JC Ilves KL Otto SP The consequences of dioecy for seed dispersal: modeling the seed-shadow handicap Evolution 2001 55 880 888 10.1554/0014-3820(2001)055[0880:TCODFS]2.0.CO;2 11430648

[B4] Wilson WG Harder LD Reproductive uncertainty and the relative competitiveness of simultaneous hermaphroditism versus dioecy American Naturalist 2003 162 220 241 10.1086/376584 12858266

[B5] Van Der Merwe M Winfield MO Arnold GM Parker JS Spatial and temporal aspects of the genetic structure of Juniperus communis populations Molecular Ecology 2000 9 379 386 10.1046/j.1365-294x.2000.00868.x 10736041

[B6] Piessens K Honnay O Nackaerts K Hermy M Plant species richness and composition of heathland relics in north-western Belgium: evidence for a rescue-effect? Journal of Biogeography 2004 31 1683 1692 10.1111/j.1365-2699.2004.01056.x

[B7] Wallis-DeVries MF Poschlod P Willems JH Challenges for the conservation of calcareous grasslands in northwestern Europe: integrating the requirements of flora and fauna Biological Conservation 2002 104 265 273 10.1016/S0006-3207(01)00191-4

[B8] Webb NR The traditional management of European heathlands Journal of Applied Ecology 1998 35 987 990

[B9] Verheyen K Adriaenssens S Gruwez R Michalczyk IM Ward LK Rosseel Y Van den Broeck A Garcia D Juniperus communis: victim of the combined action of climate warming and nitrogen deposition? Plant Biology 2009 11 49 59 19778368 10.1111/j.1438-8677.2009.00214.x

[B10] Leimu R Mutikainen P Koricheva J Fischer M How general are positive relationships between plant population size, fitness and genetic variation? Journal of Ecology 2006 94 942 952 10.1111/j.1365-2745.2006.01150.x

[B11] Severns P Inbreeding and small population size reduce seed set in a threatened and fragmented plant species, Lupinus sulphureus ssp kincaidii (Fabaceae) Biological Conservation 2003 110 221 229 10.1016/S0006-3207(02)00191-X

[B12] Michalczyk IM Opgenoorth L Luecke Y Huck S Ziegenhagen B Genetic support for perglacial survival of Juniperus communis L. in Central Europe Holocene 2010 20 887 894 10.1177/0959683610365943

[B13] Oostermeijer JGB De Knegt B Genetic population structure of the wind-pollinated, dioecious shrub Juniperus communis in fragmented Dutch heathlands Plant Species Biology 2004 19 175 184 10.1111/j.1442-1984.2004.00113.x

[B14] Provan J Beatty GE Hunter AM McDonald RA McLaughlin E Preston SJ Wilson S Restricted gene flow in fragmented populations of a wind-pollinated tree Conservation Genetics 2008 9 1521 1532 10.1007/s10592-007-9484-y

[B15] Wright S Isolation by distance under diverse systems of mating Genetics 1946 31 39 59 21009706 10.1093/genetics/31.1.39 PMC1209315

[B16] Whitlock MC McCauley DE Indirect measures of gene flow and migration: F-ST not equal 1/(4Nm+1) Heredity 1999 82 117 125 10.1038/sj.hdy.6884960 10098262

[B17] Albaladejo RG Carrillo LF Aparicio A Fernandez-Manjarres JF Gonzalez-Varo JP Population genetic structure in Myrtus communis L. in a chronically fragmented landscape in the Mediterranean: can gene flow counteract habitat perturbation? Plant Biology 2009 11 442 453 10.1111/j.1438-8677.2008.00121.x 19470115

[B18] Campbell D Duchesne P Bernatchez L AFLP utility for population assignment studies: analytical investigation and empirical comparison with microsatellites Molecular Ecology 2003 12 1979 1991 10.1046/j.1365-294X.2003.01856.x 12803646

[B19] He TH Krauss SL Lamont BB Miller BP Enright NJ Long-distance seed dispersal in a metapopulation of Banksia hookeriana inferred from a population allocation analysis of amplified fragment length polymorphism data Molecular Ecology 2004 13 1099 1109 10.1111/j.1365-294X.2004.02120.x 15078448

[B20] Cain ML Milligan BG Strand AE Long-distance seed dispersal in plant populations American Journal of Botany 2000 87 1217 1227 10.2307/2656714 10991892

[B21] Barluenga M Austerlitz F Elzinga JA Teixeira S Goudet J Bernasconi G Fine-scale spatial genetic structure and gene dispersal in Silene latifolia Heredity 2011 106 13 24 10.1038/hdy.2010.38 20389310 PMC3183859

[B22] Ouburg NJ Piquot Y Van Groenendael JM Population genetics, molecular markers and the study of dispersal in plants Molecular Ecology 1999 87 551 568

[B23] Bonin A Bellemain E Bronken Eidessen P Pompanon F Brochmann C Taberlet P How to track and assess genotyping errors in population genetics studies Molecular Ecology 2004 13 3261 3273 10.1111/j.1365-294X.2004.02346.x 15487987

[B24] Mende M Bistrom O Meichssner E Kolsch G The aquatic leaf beetle Macroplea mutica (Coleoptera: Chrysomelidae) in Europe: Population structure, postglacial colonization and the signature of passive dispersal European Journal of Entomology 2010 107 101 113

[B25] Holland BR Clarke AC Meudt HM Optimizing automated AFLP scoring parameters to improve phylogenetic resolution Systematic Biology 2008 57 347 366 10.1080/10635150802044037 18570031

[B26] Storme V Vanden Broeck A Ivens B Halfmaerten D Van Slycken J Castiglione S Grassi F Fossati T Cottrell JE Tabbener HE Lefèvre F Saintagne C S.Fluch S Krystufek V Burg K S. Bordács S Borovics A Gebhardt K Vornam B Pohl A Alba N Agúndez D Maestro C Novitol E Bovenschen J van Dam BC van der Schoot J Vosman B Boerjan W Smulders MJM *Ex-situ *conservation of Black poplar in Europe: genetic diversity in nine gene bank collections and their value for nature development Theoretical and Applied Genetics 2004 108 969 981 10.1007/s00122-003-1523-6 15067382

[B27] Meudt HM Clarke AC Almost forgotten or latest practice? AFLP applications, analyses and advances Trends in Plant Science 2007 12 106 117 10.1016/j.tplants.2007.02.001 17303467

[B28] Whitlock R Hipperson H Mannarelli M Butlin RK Burke T An objective, rapid and reproducible method for scoring AFLP peak-height data that minimizes genotyping error Molecular Ecology Resources 2008 8 725 735 10.1111/j.1755-0998.2007.02073.x 21585880

[B29] Arrigo N Tuszynski JW Ehrich D Gerdes T Alvarez N Evaluating the impact of scoring parameters on the structure of intra-specific genetic variation using RawGeno, an R package for automating AFLP scoring BMC Bioinformatics 2009 10 10.1186/1471-2105-10-33 PMC2656475 19171029

[B30] Bonin A Ehrich D Manel S Statistical analysis of amplified fragment length polymorphism data: a toolbox for molecular ecologists and evolutionists Molecular Ecology 2007 16 3737 3758 10.1111/j.1365-294X.2007.03435.x 17850542

[B31] Oddou-Muratorio S Bontemps A Klein EK Chybicki I Vendramin GG Suyama Y Comparison of direct and indirect genetic methods for estimating seed and pollen dispersal in Fagus sylvatica and Fagus crenata Forest Ecology and Management 2010 259 2151 2159 10.1016/j.foreco.2010.03.001

[B32] Garcia D Effects of seed dispersal on Juniperus communis recruitment on a Mediterranean mountain Journal of Vegetation Science 2001 12 839 848 10.2307/3236872

[B33] Hamrick JL Godt MJW L.Sherman-Broyles SL Factors influencing levels of genetic diversity in woody plant species New Forests 1992 6 95 124 10.1007/BF00120641

[B34] Petit RJ Duminil J Fineschi S Hampe A Salvini D Vendramin GG Comparative organization of chloroplast, mitochondrial and nuclear diversity in plant populations Molecular Ecology 2005 14 689 701 15723661 10.1111/j.1365-294X.2004.02410.x

[B35] Svenning JC Normand S Kageyama M Glacial refugia of temperate trees in Europe: insights from species distribution modelling Journal of Ecology 2008 96 1117 1127 10.1111/j.1365-2745.2008.01422.x

[B36] Bennett KD Tzedakis PC Willis KJ Quaternary refugia of north European trees Journal of Biogeography 1991 18 103 115 10.2307/2845248

[B37] Taberlet P Cheddadi R Quaternary refugia and persistence of biodiversity Science 2002 297 2009 2010 10.1126/science.297.5589.2009 12242431

[B38] Bennett KD Boreham S Sharp MJ Switsur VR Holocene history of environment, vegetation and human settlement on Catta Ness, Lunnasting, Shetland Journal of Ecology 1992 80 241 273 10.2307/2261010

[B39] Cottrell J Krystufek V Tabbener H Milner A Connolly T Sing L Fluch S Burg K Lefèvre F Achard P Bodrács S Gebhardt K Vornam B Smulders MJM Vanden Broeck AH Van Slycken J Storme V Boerjan W Castiglione S Fossati T Alba N Agúndez D Maestro C Novitol E Bovenschen J van Dam BC Postglacial migration of Populus nigra L.: lessons learnt form chloroplast DNA Forest Ecology and Management 2004 206 71 90

[B40] Petit RJ Brewer S Bordács S Burg K Cheddadi R Coart E Cottrell J Csaikl UM Van Dam B Espinel S Fineschi S Finkeldey R Glaz I Goicoechea PG Jensen JS König AO Lowe AJ Madsen SF Mátyás G Munro RC Popescu F Slade D Tabbener H de Vries SGM Ziegenhagen B de Beaulieu J-L Kremer A Identification of refugia and post-glacial colonisation routes of European white oaks based on chloroplast DNA and fossil pollen evidence Forest Ecology and Management 2002 156 49 74 10.1016/S0378-1127(01)00634-X

[B41] Mao K Hao G Liu J Adams RP Milne RI Diversification and biogeography of *Juniperus *(Cupressaceae): variable diversification rates and multiple intercontinental dispersals New Phytologist 2010 188 254 272 10.1111/j.1469-8137.2010.03351.x 20561210

[B42] Lowe AJ Boshier D Ward M Bacles CFE Navarro C Genetic resource impacts of habitat loss and degradation; reconciling empirical evidence and predicted theory for neotropical trees Heredity 2005 95 255 273 10.1038/sj.hdy.6800725 16094300

[B43] Mathiasen P Rovere AE Premoli AC Genetic structure and early effects of inbreeding in fragmented temperate forests of a self-incompatible tree, Embothrium coccineum Conservation Biology 2007 21 232 240 10.1111/j.1523-1739.2006.00565.x 17298529

[B44] Verheyen K Schreurs K Vanholen B Hermy M Intensive management fails to promote recruitment in the last large population of Juniperus communis (L.) in Flanders (Belgium) Biological Conservation 2005 124 113 121 10.1016/j.biocon.2005.01.018

[B45] Honnay O Bossuyt B Prolonged clonal growth: escape route or route to extinction? Oikos 2005 108 427 432 10.1111/j.0030-1299.2005.13569.x

[B46] Ward LK The conservation of juniper. Present status of juniper in southern England Journal of Applied Ecology 1973 10 165 188 10.2307/2404724

[B47] Adams RP Junipers of the World: The genus Juniperus 2008 Vancouver: Trafford Publishing

[B48] Kollmann J Ausbreitungsökologie endozoochorer Gehölzarten 1994

[B49] van der Meijden R Odé B Groen CLG Witte JPM Bal D Bedreigde en kwetsbare vaatplanten in Nederland. Basisrapport met voorstel voor de Rode lijst Gorteria 2000 26 85 208

[B50] Clifton SJ Ward LK Ranner DS The status of juniper (Juniperus communis L.) in north-east England Biological Conservation 1997 79 67 77 10.1016/S0006-3207(96)00101-2

[B51] Saintenoy-Simon J Barbier Y Delescaille LM Dufrêne M Gathoye JL Verté P Première liste des espèces rares, menacées et protégées de la Région Wallonne (Ptéridophytes et Spermatophytes) 2006 Version 1 (7/3/2006)

[B52] Van Landuyt W Vanhecke L Hoste I Van Landuyt W, Hoste I, Vanhecke L, Vercruysse W, Van den Bremt P, De Beer D Rode Lijst van de vaatplanten van Vlaanderen en het Brussels 2006 INBO and Nationale Plantentuin van België, Brussel

[B53] Thomas PA El-Barghathi M Polwart A Biological flora of the British isles: Juniperus communis L Journal of Ecology 2007 95 1404 1440 10.1111/j.1365-2745.2007.01308.x

[B54] Cottam G Curtis JT The use of distance measures in phytosociological sampling Ecology 1956 37 451 460 10.2307/1930167

[B55] Piessens K Hermy M Does the heathland flora in north-western Belgium show an extinction debt? Biological Conservation 2006 132 382 394 10.1016/j.biocon.2006.04.032

[B56] Adriaenssens S Baeten L Crabbe S Verheyen Kris Evolutie (1985-2006) en toekomst van de jeneverbes (Juniperus communis L.) in de provincie Limburg 2006 Universiteit Gent & Likona 82

[B57] Miller AL McDonald MB, Kwong FY Tetrazolium testing for flower seeds Flower seeds; Biology and Technology 2004 Wallingford: CABI Publishing 229 31021829164

[B58] Vos P Hogers R Bleeker M Reijans M van de Lee T Hornes M Frijters A Pot J Peleman J Kuiper M Zabeau M AFLP: a new technique for DNA fingerprinting Nucleic Acids Research 1995 23 4407 4414 10.1093/nar/23.21.4407 7501463 PMC307397

[B59] Coart E Van Glabeke S Petit RJ Van Bockstaele E Roldan-Ruiz I Range wide versus local patterns of genetic diversity in hornbeam (Carpinus betulus L.) Conservation Genetics 2005 6 259 273 10.1007/s10592-004-7833-7

[B60] Sokal RR Michener CD A Statistical Method for Evaluating Systematic Relationships The University of Kansas Scientific Bulletin 1958 38 1409 1438

[B61] Van De Peer Y De Wachter R TREECON for Windows: a software package for the construction and drawing of evolutionary trees for the Microsoft Windows environment Computer Applications in the Bioscience 1994 10 569 570 10.1093/bioinformatics/10.5.569 7828077

[B62] Duchesne P Bernatchez L AFLPOP: a computer program for simulated and real population allocation, based on AFLP data Molecular Ecology Notes 2002 2 380 383 10.1046/j.1471-8286.2002.00251.x

[B63] Bossard M Feranec J Otahel J CORINE Land Cover Technical Guide 2000 European Environment Agency, Technical report No. 40 http://www.eea.europa.eu

[B64] Paetkau D Calvert W Stirling I Strobeck C Microsatellite analysis of population-structure in Canadian polar bears Molecular Ecology 1995 4 347 354 10.1111/j.1365-294X.1995.tb00227.x 7663752

[B65] Huff DR Peakall R Smouse PE Rapd variation within and among natural-populations of outcrossing buffalograss [Buchloe-Dactyloides (Nutt) Engelm] Theoretical and Applied Genetics 1993 86 927 934 10.1007/BF00211043 24193999

[B66] Wright S The genetical structure of populations Annual Eugenetics 1951 15 323 354 10.1111/j.1469-1809.1949.tb02451.x 24540312

[B67] Peakall R Smouse PE GENALEX 6: genetic analysis in Excel. Population genetic software for teaching and research Molecular Ecology Notes 2006 6 288 295 10.1111/j.1471-8286.2005.01155.x PMC3463245 22820204

[B68] Corander J Marttinen P Siren J Tang J Enhanced Bayesian modelling in BAPS software for learning genetic structures of populations BMC Bioinformatics 2008 9 10.1186/1471-2105-9-539 PMC2629778 19087322

[B69] Rousset F Genetic differentiation and estimation of gene flow from F-statistics under isolation by distance Genetics 1997 145 1219 1228 9093870 10.1093/genetics/145.4.1219 PMC1207888

[B70] Hardy OJ Estimation of pairwise relatedness between individuals and characterization of isolation-by-distance processes using dominant genetic markers Molecular Ecology 2003 12 1577 1588 10.1046/j.1365-294X.2003.01835.x 12755885

[B71] Hardy OJ Vekemans X SPAGEDi: a versatile computer program to analyse spatial genetic structure at the individual or population levels Molecular Ecology Notes 2002 2 618 620 10.1046/j.1471-8286.2002.00305.x

[B72] Ehrich D AFLPDAT: a collection of R functions for convenient handling of AFLP data Molecular Ecology Notes 2006 6 603 604 10.1111/j.1471-8286.2006.01380.x

[B73] Vekemans X Beauwens M Lemaire M Roldán-Ruiz I Data from amplified fragment length polymorphism (AFLP) markers show indication of size homoplasy and of a relationship between degree of homoplasy and fragment size Molecular Ecology 2002 11 139 151 10.1046/j.0962-1083.2001.01415.x 11903911

[B74] Zhivotovsky LA Estimating population structure in diploids with multilocus dominant DNA markers Molecular Ecology 1999 8 907 913 10.1046/j.1365-294x.1999.00620.x 10434412

[B75] Dasmahapatra KK Lacy RC Amos W Estimating levels of inbreeding using AFLP markers Heredity 2008 100 286 295 10.1038/sj.hdy.6801075 17987055

